# Enhanced peripheral visual processing in congenitally deaf humans is supported by multiple brain regions, including primary auditory cortex

**DOI:** 10.3389/fnhum.2014.00177

**Published:** 2014-03-26

**Authors:** Gregory D. Scott, Christina M. Karns, Mark W. Dow, Courtney Stevens, Helen J. Neville

**Affiliations:** ^1^Brain Development Lab, Department of Psychology, University of OregonEugene, OR, USA; ^2^Division of Pulmonary and Critical Care Medicine, Department of Medicine, Oregon Health and Science UniversityPortland, OR, USA; ^3^Department of Psychology, Willamette UniversitySalem, OR, USA

**Keywords:** visual attention, deaf, human, fMRI, Heschl's gyrus, auditory cortex

## Abstract

Brain reorganization associated with altered sensory experience clarifies the critical role of neuroplasticity in development. An example is enhanced peripheral visual processing associated with congenital deafness, but the neural systems supporting this have not been fully characterized. A gap in our understanding of deafness-enhanced peripheral vision is the contribution of primary auditory cortex. Previous studies of auditory cortex that use anatomical normalization across participants were limited by inter-subject variability of Heschl's gyrus. In addition to reorganized *auditory* cortex (cross-modal plasticity), a second gap in our understanding is the contribution of altered modality-specific cortices (visual intramodal plasticity in this case), as well as supramodal and multisensory cortices, especially when target detection is required across contrasts. Here we address these gaps by comparing fMRI signal change for peripheral vs. perifoveal visual stimulation (11–15° vs. 2–7°) in congenitally deaf and hearing participants in a blocked experimental design with two analytical approaches: a Heschl's gyrus region of interest analysis and a whole brain analysis. Our results using individually-defined primary auditory cortex (Heschl's gyrus) indicate that fMRI signal change for more peripheral stimuli was greater than perifoveal in deaf but not in hearing participants. Whole-brain analyses revealed differences between deaf and hearing participants for peripheral vs. perifoveal visual processing in extrastriate visual cortex including primary auditory cortex, MT+/V5, superior-temporal auditory, and multisensory and/or supramodal regions, such as posterior parietal cortex (PPC), frontal eye fields, anterior cingulate, and supplementary eye fields. Overall, these data demonstrate the contribution of neuroplasticity in multiple systems including primary auditory cortex, supramodal, and multisensory regions, to altered visual processing in congenitally deaf adults.

## Introduction

A number of studies report alterations in visual performance following deafness (Bavelier and Neville, 2002; reviewed in Bavelier et al., 2006). In general, deaf individuals show enhanced motion detection, visual orienting, and selective attention in peripheral but not central visual fields (Neville and Lawson, 1987b; Loke and Song, 1991; Bosworth and Dobkins, 2002a; Stevens and Neville, 2006) (but see Bottari et al., 2011). Several studies have examined the neural substrates supporting these enhancements. In deaf cats, visual enhancements rely on specific regions of the auditory cortex serving a homologous function (Lomber et al., 2010). Data from adult humans suggest that both intramodal plasticity and supramodal cortex also contribute to enhanced peripheral visual processing. For example, neuroimaging studies of visual processing in congenitally deaf humans show increased responses in visual cortex (e.g., motion sensitive MT+/V5) and attention-related brain networks in tasks using peripheral visual stimuli (Bavelier et al., 2001; Bavelier and Neville, 2002). Previous studies suggest this these alterations may be partially accounted for by increased attention to the periphery in deaf participants (Neville et al., 1983; Neville and Lawson, 1987a,b; Bavelier et al., 2000, 2001).

### Cross-modal neuroplasticity of primary auditory cortex

A key question in cross-modal plasticity is whether congenital deafness affects the organization of low-level primary sensory cortex or whether these specialized brain regions are exempt from neuroplasticity. Many studies testing this question have reported no or non-specific responses to visual stimuli in primary auditory cortex (Vachon et al., 2013; see Bavelier et al., 2006 for a review). In contrast, a few studies have suggested cross-modal plasticity of primary auditory cortex (visual motion stimuli: Finney et al., 2001; vibrotactile stimuli: Auer et al., 2007). The main limitation of these studies is that the site of human primary auditory cortex, Heschl's gyrus, is highly variable in its morphology across individuals and approaches that use spatial averaging across participants and identification based on atlases suffer from poor localization and potential loss of power due to low spatial overlap across participants. We recently addressed this using individual anatomically-defined Heschl's gyrus regions of interest and showed that Heschl's gyrus responds to both touch and vision in congenitally deaf adults, and further, the cross-modal responses in primary auditory cortex were larger contralateral to stimulation (Karns et al., 2012).

It is important to begin comparing findings in humans with findings in animal models that allow for regional specificity in terms of cytoarchitecture and function. For example some animal studies indicate that primary auditory cortex or closely related regions respond to vision and somatosensation in early deafness (Allman et al., 2009; Meredith et al., 2009; Lomber et al., 2010; Meredith and Lomber, 2011), while other studies report deaf primary auditory cortex does not respond to vision (Kral et al., 2003). Comparisons across human and animal models are complicated by imprecise delineation of low-level auditory cortex in humans (Hackett, 2011) but progress is being made. For example, according to a recent tonotopic fMRI study the anterior aspect of Heschl's likely corresponds mainly to human A1, while the posterior aspect corresponds to human area R (Da Costa et al., 2011). Both A1 and R are core auditory regions in the macaque monkey (Hackett, 2011). Although tonotopy is not possible in deaf populations, in the current study we parcellate Heschl's gyrus into its anatomical anterior and posterior divisions (putative analog to primate areas A1 and R, respectively) to contribute to cross-species comparisons and to test the hypothesis that enhanced peripheral vision in deafness is reflected by enhanced visual responses of peripheral vs. perifoveal visual stimulation in deaf primary auditory cortex.

### Adaptation and plasticity in other brain regions

Although a region of interest approach is important for assessing plasticity in brain areas with high anatomical variability like Heschl's gyrus, a strength of human fMRI is the ability to obtain whole brain coverage with reasonable spatial specificity. Using these whole-brain approaches, neuroimaging studies of motion- and attention-related visual processing in deaf humans have revealed increased responses in brain regions involved as both the *sources* of attention modulation (i.e., FEF, PPC, post-STS) as well as likely *sites* of attentional modulation (MT+, V1/V2, V3a), including higher-level extrastriate visual cortex (e.g., MT+/V5) (Bavelier et al., 2000, 2001). However, it is difficult to determine whether these enhancements occur with simple visual stimuli presented in the periphery or whether such plasticity requires higher-level stimulus properties (e.g., shape, motion) or high attention load. To address these questions, we performed whole brain group analyses in deaf and hearing participants comparing brain responses to simple point-light stimuli presented in the peripheral vs. perifoveal visual field.

In summary, the primary goal of the current study was to determine whether primary auditory cortex supports enhanced visual processing of the visual periphery in profoundly, congenitally deaf adults. In addition, we sought to determine whether multisensory and/or supramodal cortices support enhanced peripheral visual processing in congenitally deaf humans when simple visual stimuli are presented in a low-level visual detection task. To do this, in the present study we combined region-of-interest, individual parcellation techniques to measure responses within primary auditory cortex (Heschl's gyrus), along with whole brain group analysis comparing deaf and hearing participants to perifoveal vs. peripheral visual stimulation using a target detection task.

## Materials and methods

### Participants

Ten congenitally and profoundly deaf adults (six females) and seven hearing adults (five females) participated in the current study. Participant ages ranged from 19- to 45-years-old (Deaf: mean age 30 years ± 7.6, range 19–45, Hearing: mean age 30 years ± 10.6, range 20–45). One deaf participant was excluded from final analysis due to motion-related artifacts. All deaf participants reported being profoundly and congenitally deaf due to heredity. Audiogram data, available from four of the nine deaf participants, confirmed profound deafness, with a mean hearing level of 100 dB (range 91–110 dB). All deaf participants were also native users of American Sign Language. All research was performed with the written informed consent of participants and in compliance with the Human Subjects Institutional Review Board at the University of Oregon.

### Apparatus

**Figure 1:**
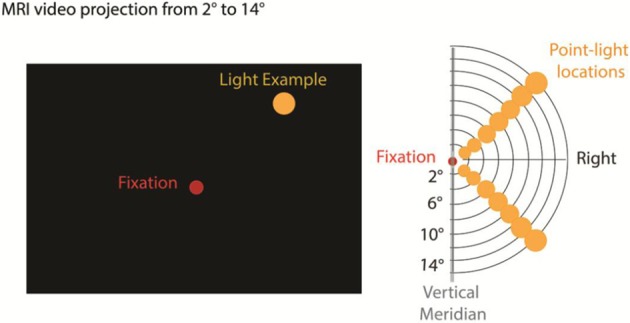
**Stimulus presentation and Apparatus**. We used a standard MRI video projection system, back projected to a screen viewed by the participants through a monitor. A red fixation point was displayed continuously at center and single point yellow light stimuli were presented in the upper or lower right visual field at polar eccentricities ranging from 2 to 15°. Standard stimuli (85%) were flashed at 8 Hz and target stimuli requiring a button press (15%) were flashed at 14 Hz.

An MRI video projection apparatus was used to present single, yellow discrete disk stimuli on a video screen at eccentricities of 2, 4, 7, 9, 11, 13, and 15° (Figure 1). Stimuli were presented via an InFocus LP350 video system viewed by the subject through a mirror mounted on the MRI head coil. A dim red light was continuously present at fixation (Figure 1). All stimuli were presented along polar angle radials 45° above and below the horizontal meridian in the right visual field. We focused on the right visual field due to fMRI time constraints; the left eye was patched to reduce visual fatigue. Similar discrete stimulus presentation has been used in previous retinotopic experiments (Tootell et al., 1998a,b; Di Russo et al., 2001; Bridge et al., 2005; Lu et al., 2005). Stimuli flashed at 8 Hz (standards: 85% probable) or 14 Hz (targets: 15% probable) for 3.2 s before changing location in a pseudo-random sequence optimizing the visual distance of sequential stimuli. Stimulus timing was controlled by Presentation software (Neurobehavioral Systems).

### Task

The participants' task was to detect visual stimuli that flickered at a faster rate (14 Hz) than standard stimuli (8 Hz). Participants pressed a keypad in response to the target stimuli while maintaining central fixation and attending to the entire right visual field. Prior to scanning, participants practiced the task using a computer simulation outside the magnet room until they were able to accurately detect every target. During the scan, researchers monitored subject compliance to response and fixation instructions using on-line response checking and an ASL 5000/LRO infrared eye tracker (Applied Science Laboratories), although behavioral responses were not able to be recorded due to an error in the experimental apparatus.

### MRI data acquisition and analysis

MRI data were acquired using a Siemens Allegra (3-Tesla) magnetic resonance imaging system (Siemens Medical Systems) equipped with a transmit/receive volume head coil. Head movement was restricted using a vacuum pillow and side cushioning. Blood oxygenation level dependent (BOLD) images were acquired with an echo-planar imaging sequence (32 slices, interleaved acquisition, 3 mm^2^ in-plane resolution, 3 mm thickness, no inter-slice gap, *TR* = 2000 ms, *TE* = 30 ms) with a 192 mm field of view. Axial slices were oriented in the transverse plane roughly parallel with the base of the brain. Each functional scan lasted for 5.5 min and consisted of 165 acquisitions. Each scanning session was composed of eight independent functional runs. For anatomical localization, high-resolution (1 mm^3^) T1-weighted images of the whole brain were acquired, using a 3-D Magnetization-Prepared Rapid Acquisition Gradient echo sequence (3-D MP-RAGE, 50% distance factor, *TR* = 2500 ms, *TE* = 4.38 ms). The total time a participant spent in the scanner per experimental session was typically 1–1.5 hours.

Image processing was performed using FEAT, a part of the FMRIB Software Library (FSL) analysis suite (Smith et al., 2004). Functional image preprocessing included: motion correction using MCFLIRT, EPI non-brain removal using BET, 8 mm smoothing, high-pass temporal filtering (cutoff = 0.01 Hz) and affine coregistration to the anatomical scan (Jenkinson et al., 2002; Smith, 2002). Each participant's anatomical volume was stripped of non-brain voxels and coregistered to the MNI 152 subject brain template using a 12° of freedom affine model in FLIRT, FSL's linear coregistration tool (Jenkinson and Smith, 2001; Jenkinson et al., 2002). Brain extractions, where portions of cortex were inadvertently removed or remaining skull were identified via manual inspection and reprocessed following adjusted BET search parameters and/or manual segmentation using Space Software (http://lcni.uoregon.edu/~dow/Space_program.html). Task-related regressors were modeled at each visual-field location with each visual stimulus represented by a box-car (duration 3.125 s) that was then convolved with the FSL canonical hemodynamic response function (Gamma function, delay = 6 s, standard deviation = 3 s).

### Individual heschl's gyrus region of interest (ROI) analysis

To investigate whether primary auditory cortex was sensitive to increasing eccentricity and whether any variation was greater in deaf than hearing participants, we performed anatomical parcellations of Heschl's gyrus in individual brains following the methods described in our previous study (Karns et al., 2012). A caveat in both studies is that estimating primary auditory cortex boundaries based on gross anatomical landmarks may include a small portion of other auditory cortex fields (Morosan et al., 2001; Da Costa et al., 2011) however tonotopic methods are also susceptible to this caveat (Da Costa et al., 2011) and cannot be used with profoundly deaf participants. Because of considerable morphological variability across participants, anatomical segmentation is more precise than spatial coregistration and atlas-based determinations of Heschl's gyrus in group-analyses (Karns et al., 2012). Each Heschl's gyrus was parcellated by hand by raters blind to deaf or hearing status on a structural volume (T1-weighted MPRAGE) coregistered and resampled to the 2 × 2 × 2 mm FSL standard brain (MNI 152); ROIs were drawn using Space Software (http://lcni.uoregon.edu/~dow/Space_program.html). On sagittal planes, an initial boundary of Heschl's gyrus was drawn and in cases where there was a double Heschl's gyrus, both anterior and posterior gyri were included in the parcellation. These boundaries were projected onto coronal planes and adjusted if the gyrus was visible in a cross section at either sagittal or coronal orientation. The boundaries were also checked in projection on the axial planes, where voxels with low neighborhood support were excluded and voxels with high neighborhood support included. The individual Heschl's gyri were divided into Anterior and Posterior subdivisions by positioning a vertical plane oriented along the principle axis of voxel centers to allow comparison to tonotopic functional neuroimaging demonstrating human primary cortical areas A1 and R respect anatomical boundaries of anterior and posterior Heschl's gyrus, respectively. A second rater validated all parcellations and if there were any discrepancies the brain was re-entered into the set with a new subject number to be parcellated again by the original rater, who was unaware that they were re-rating a brain that they had previously parcellated. Any remaining disagreements in parcellation decisions were discussed and a final parcellation decision was made based on the protocol described above. The parameter estimates from all un-thresholded voxels within the boundary were extracted for the contrast between peripheral and perifoveal visual stimulation and scaled to percent signal change. We performed a repeated measures ANOVA (2 Hemispheres [Contralateral-Left, Ipsilateral-Right] × 2 Heschl's Gyrus Subregions [Anterior, Posterior]) with Group (Deaf, Hearing) as a between subjects factor.

### Peripheral visual processing: whole-brain group analyses

Group analyses were performed using the FSL analysis suite. Individual anatomical volumes were aligned to the Montreal Neurological Institute average template (ICBM152) using FLIRT affine coregistration. The resultant transformation was applied to all spatially smoothed EPI volumes to standardize functional data. Group analyses were performed using FLAME mixed effects error propagation with statistical thresholding reported for Z > 2.3 and corrected for multiple comparisons using a cluster probability thresholding of *p* = 0.05 (Worsley et al., 1992; Beckmann et al., 2003; Woolrich et al., 2004). To test whether differences between deaf and hearing increased as eccentricity increased, the contrasts for each experiment were 11–15° vs. 2–7°. Further analyses were conducted in each group alone.

## Results

### Heschl's gyrus region of interest

**Figure 2:**
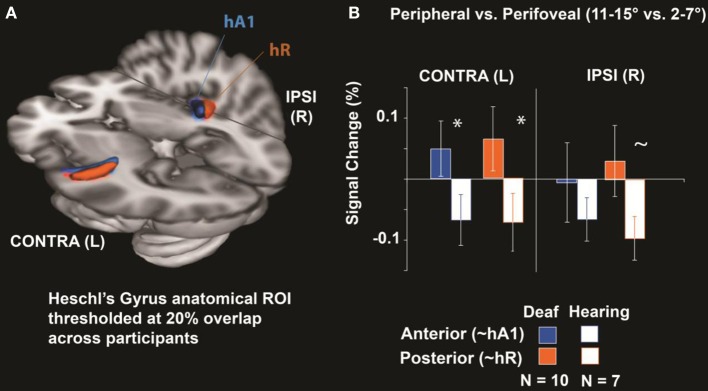
**Heschl's Gyrus Region of interest analysis. (A)** Anatomical Heschl's gyrus ROIs drawn on individual structural brain images divided along the first principal component axis into an anterior and posterior subregion to approximate primate primary-auditory areas A1 and R respectively (as defined tonotopically in hearing adults; Da Costa et al., 2011), shown here thresholded at 20% overlap across participants. **(B)** Differences between peripheral (11–15°) and perifoveal locations (2–7°) were significantly larger in deaf than hearing participants for anterior and posterior Heschl's gyrus contralateral to visual stimulation (^*^p < 0.05) and tended to be larger in deaf participants posterior Heschl's gyrus ipsilateral to stimulation (~ *p* < 0.10). Error bars represent ± s.e.m.

Percent signal change in anatomically-defined Heschl's gyrus was measured for the 11–15° vs. 2–7° contrast. Heschl's gyrus was divided into an anterior and posterior subregion (Figure 2A) to approximate primate primary-auditory areas A1 and R respectively (Da Costa et al., 2011). As shown in Figure 2B, hearing individuals had decreased signal in Heschl's gyrus for peripheral compared to perifoveal stimulation—represented as a negative signal change. In contrast, deaf individuals showed a positive signal increase for peripheral visual stimulation relative to perifoveal. Differences between deaf and hearing manifested as a Subregion × Group Interaction [*F*_(1, 15)_ = 6.6, *p* = 0.02]. Follow up *t*-tests indicated that the deaf had a larger signal difference between peripheral and perifoveal locations than the hearing in Contralateral Anterior Heschl's Gyrus [*T*_(15)_ = 1.81, 0.045, one-tailed] and Contralateral Posterior Heschl's Gyrus [*T*_(15)_ = 1.83, *p* = 0.044] and tended to be larger in the Ipsilateral Posterior subregion [*T*_(15)_ = 1.66, *p* = 0.059]. These results indicate an increase in HG signal with increasing visual eccentricity from 2–7° to 11–15° in the deaf but not hearing participants.

### Group analyses

**Figure 3:**
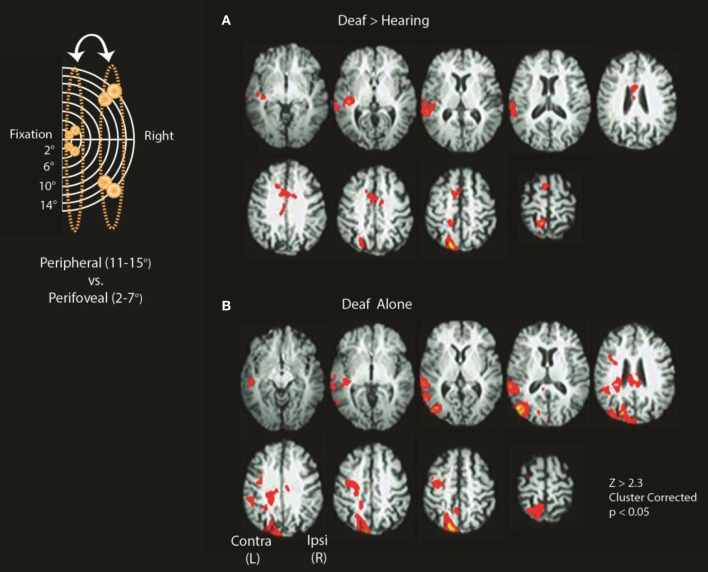
**Differences between deaf and hearing for peripheral vs. perifoveal stimulation**. Inset shows a schematic location of stimuli included in the contrasts (11–15° vs. 2–7°). **(A)** Deaf > Hearing and **(B)** Deaf alone. See Tables 1, 2 for a summary of significant clusters and corresponding atlas-based descriptions.

Group-level analyses were performed to identify regions that showed differential recruitment to peripheral vs. perifoveal visual presentation. As shown in Figure 3A and detailed in Table 1, for peripheral 11–15° vs. perifoveal 2–7° stimuli, deaf participants showed greater activation than hearing participants in left superior-temporal auditory and multisensory cortex as well as brain regions that have also been associated with attention [left posterior parietal cortex (PPC), and anterior cingulate/SEF] (*Z* > 2.3, *p* < 0.05 corrected).

**Table 1:** **Periphery (11–15) vs. central (2–7): deaf > hearing**.

**Hemi**	Z^*^	**Coordinates**			Harvard oxford atlas^**^	Juelich^**^
		***x***	***y***	***z***		
L	3.88	−18	−80	46	71% Superior LOC	16% SPL
L	3.64	−16	−40	50	11% Precuneus, 10% posterior cingulate	27% SPL
L	3.51	−16	−40	54	26% Postcentral G.	28% Prim. SS, 28% Prim. motor
L	3.44	−22	−62	56	42% Superior LOC	77% SPL 7A
L	3.41	−12	−52	64	22% SPL, 19% precuneous, 14% postcentral G.	54% SPL 5, 38% SPL 7A
L	3.17	−16	−48	56	18% Postcentral G., 12% SPL	40% SPL 5, 24% SPL 7A
L	3.52	−6	10	62	25% Superior frontal G.	48% Premotor BA6
R	3.1	2	4	36	92% Anterior cingulate	
L	3.07	−6	4	22		69% Callosal body
L	3.03	−2	2	42	71% Anterior cingulate	6% Premotor BA6
L	3.01	−6	−12	28	9% Anterior cingulate	83% Callosal body
	3.01	0	6	22	3% Anterior cingulate	85% Callosal body
L	3.57	−66	−34	0	43% pMTG, 43% pSTG	10% Inferior parietal lobule
L	3.44	−64	−20	8	41% Planum temporale, 27% pSTG	17% Secondary SS
L	3.35	−52	−30	6	31% Planum temporale	16% Prim. auditory
L	3.32	−58	−38	6	58% pSTG	
L	3.31	−46	−26	−4	27% Heschl's G., 24% planum temporale	40% Prim. auditory
L	3.26	−48	−26	2	22% Planum temporale, 8% Heschl's G.	27% Prim. auditory

* Survives cluster correction, Z > 2.3, p < 0.05.

** Most probable locations.

Abbreviations: L, left; LOC, lateral occipital cortex; SS, somatosensory; SPL, superior parietal lobule; pMTG, posterior middle temporal gyrus; pSTG, posterior superior temporal gyrus; Prim., primary; Hemi., hemisphere; G., gyrus.

In cluster-corrected analyses performed separately within each group, the hearing participants did not show any regions with a larger response to peripheral (11–15°) vs. perifoveal (2–7°) stimuli. In contrast, deaf participants recruited higher order visual areas (contralateral MT+/V5), contralateral auditory, and multisensensory superior temporal cortex, and attention-related brain regions (left PPC, left FEF) as shown in Figure 3B and Table 2. Within each group, we did not observe significant signal increase in anterior calcarine sulcus.

**Table 2:** **Periphery (11–15) vs. central (2–7): deaf alone**.

**Hemi**	Z^*^	**Coordinates**			Harvard oxford atlas^**^	Juelich^**^
		***x***	***y***	***z***		
L	4.43	−50	−86	2	17% Inferior LOC	
L	4.24	−16	−80	48	66% Superior LOC	20% SPL 7P
L	4.24	−50	−74	8	1% LOC	
L	4.09	−22	−86	28	48% Superior LOC, 10% occipital pole	
L	4.05	−10	−86	44	42% Superior LOC	19% SPL
L	4	−46	−80	16	49% Superior LOC, 34% inferior LOC	44% Inferioe parietal lobule (PGp)

* Survives cluster correction, Z > 2.3, p < 0.05.

** Most probable locations.

Abbreviations: L, left; LOC, lateral occipital cortex; SPL, superior parietal lobule; G., gyrus.

## Discussion

The present study reveals a network of brain regions exhibiting enhanced responsiveness to peripheral visual stimuli in profoundly, genetically, and congenitally deaf adults. It is particularly noteworthy, given existing controversies (see Bavelier and Neville, 2002; Karns et al., 2012), that we found that individual anatomically-defined Heschl's gyrus regions, the site of human primary auditory cortex, showed a reliable increased response to peripheral vs. perifoveal visual stimulation in deaf participants. Greater signal in Heschl's gyrus occurred for both the anterior and posterior division (putative analog to primate A1 and R, respectively). Additionally, we found that regions supporting higher order visual processing (MT+/V5), as well as well as multimodal areas implicated in multisensory integration and attention (STS, PPC, anterior cingulate/SEF, and FEF) (Levanen et al., 1998; McCullough et al., 2005) which showed greater signal change for peripheral visual processing in deaf participants. Taken together, these data suggest that neuroplasticity supporting enhanced peripheral visual processing in congenital deafness involves recruitment of low level sensory cortex that has been deprived of its default sensory modality, as well as network-level recruitment of cortices involved in attention.

### Primary auditory cortex

Evidence from complimentary methodology indicates that early deafness affects the anatomy of Heschl's gyrus. Structurally, auditory cortex in the deaf exhibits significantly less white matter, larger gray matter-white matter ratios, and a steeper increase in the ascending ramus of the left posterior sylvian fissure compared to hearing participants (Emmorey et al., 2003; Meyer et al., 2007; Shibata, 2007) as well as microstructural differences in fractional anisotropy, radial diffusivity, and axial diffusivity (Kim et al., 2009; Li et al., 2011; Miao et al., 2013). These findings alone might imply that Heschl's gyrus would be less-responsive to stimulation in general due to atrophy and functional analyses such as those in the present study are critical to determine the extent to which these regions demonstrate cross-modal neuroplasticity.

Due to methodological limitations, until recently it was not clear whether Hecshl's gyrus responded to cross-modal stimulation in deafness. Studies of altered organization in deaf participants in the visual modality have generally reported cross-modal altered organization caudal to rather than overlapping Heschl's gyrus (reviewed in Bavelier et al., 2006) and studies that did report primary auditory cross-modal responses in deafness did not define Heschl's gyrus individually (Fine et al., 2005; Auer et al., 2007). It is important to define Heschl's gyrus individually due to it's high level of anatomical variability. More specifically, using a group analysis with low spatial overlap could lead to an inability to detect true responses at the individual level. Conversely, low anatomical precision due to using atlas-based localization of Heschl's gyrus could lead to misattribution of neighboring superior temporal cortex to Heschl's gyrus responses. The role of primary auditory cortex in crossmodal plasticity was recently highlighted in our report showing somatosensory responses in anatomically-defined Heschl's gyrus of deaf participants, with the response amplitude correlated with a somatosensory double-flash illusion in the deaf (Karns et al., 2012). In animal studies, tracing also demonstrates a critical period for auditory cortex reorganization associated with the somatosensory modality (Allman et al., 2009) with potentially intact auditory connectivity in deaf ferrets (Meredith et al., 2012). In other words, in spite of atypical structure of Heschl's gyrus in deafness, it responds to cross-modal stimulation and plays a role in neuroplasticity and cortical reorganization.

The current study expands upon our previous work that demonstrates a functional link between the auditory cortex and cross-modal processing in the deaf. Although our recent report demonstrated visual responses in Heschl's gyrus (Karns et al., 2012), those responses were compared to a resting fixation baseline and thus stimulus properties and attention requirements were different across the contrast. Other reports in addition to our own also support the idea that visual responses in temporal cortices reflect increased signal relative to an implicit baseline rather than differential deactivation (Vachon et al., 2013). Given these findings, in the current study we focused our experimental design on comparing perifoveal stimulation to peripheral stimulation with target detection and visual stimulation at all stimulus locations to elucidate differences between perifoveal and peripheral stimulation. In addition, MRI background sounds are inherent to the MRI technique and cannot be matched between deaf and hearing groups, but requiring attention to the task for both peripheral and perifoveal stimulation may mitigate the potential confound of hearing people paying more attention to scanner noises during resting fixation. In spite of these methodological differences between our previous report and the present one, we replicate several observations in this non-overlapping sample of congenitally deaf adults. First, the magnitude of the unimodal visual response in Heschl's gyrus for deaf participants was similar across the two studies (~0.1% signal change) even though the comparisons were different (peripheral 11–15° vs. perifoveal 2–7° target detection in the current experiment and far peripheral 45° visual stimulation vs. central resting fixation in our previous experiment). It is important to note that somatosensory responses in our previous study were considerably larger (~0.3% signal change) than visual stimulation. Both studies showed a more robust response in the Heschl's gyrus contralateral to visual stimulation, but a limitation is that stimulation in both studies always occurred on the right and future studies need to address whether this generalizes to left visual field stimulation. Nonetheless, the similarities across the results from two studies that used different stimulation methods and different participants is reassuring.

If visual stimulation recruits primary auditory cortex in deaf participants, particularly for the peripheral visual field, it is important to consider how this altered organization arises. While traditionally assigned to low-level auditory processing, more recently primary auditory cortex has also been linked to multisensory functionality and neuroplasticity in humans (see Levanen et al., 1998; Fine et al., 2005; Schroeder and Foxe, 2005; Musacchia and Shroeder, 2009). Stabilization of cross-modal connections is one potential mechanism; if cross-modal connections are typical in the auditory cortex of hearing individuals these connections could increase when acoustic input is reduced.

Although in the present study we have focused on primary auditory cortex, an important question is the degree to which low-level visual or primary visual cortex is differentially recruited for peripheral visual processing in congenitally deaf adults. Provocative EEG data may demonstrate differences between deaf and hearing individuals in early visual cortical ERP components 80 ms after stimulus onset (Bottari et al., 2011) but the degree to which preparatory potentials influence early-latency visual responses remain to be elucidated. While our group-level analyses have not shown differences in primary visual cortex, the calcarine sulcus, like Heschl's gyrus, has high variability across individuals and approaches with more anatomical specificity may show differences in deafness. Future studies will address this deficiency.

### Multisensory and higher order brain regions

In addition to enhancements in lower-level auditory areas, deaf participants in the present study displayed a crossmodal neuroplasticity in a number of regions including MT+/V5, STS, PPC, and anterior cingulate/SEF and FEF. Previous studies have linked these regions to visuospatial attention (Nobre et al., 1997; Buchel et al., 1998; Beauchamp et al., 2001; Serences et al., 2005; Saygin and Sereno, 2008) as well as multisensory integration (Hagen et al., 2002; Laurienti et al., 2003; Beauchamp et al., 2004; Blake et al., 2004; Woldoff et al., 2004; Mulette-Gillman et al., 2005; Mukai et al., 2007). Previous research also showed that MT+/V5 exhibits increased activation in the deaf in response to attended stimuli particularly in the periphery (Bavelier et al., 2000, 2001) and STS and PPC exhibits enhanced recruitment in deaf participants with attention (Bavelier et al., 2000, 2001). Our data shows that this network is differentially recruited for peripheral stimulation in deaf participants despite use of simple visual stimuli; this suggests that the common function of these brain regions in visuospatial attention and multisensory integration may be a determining factor in the enhancements they display in the deaf.

Previous research in animal models has shown that there can be high specificity in terms of which brain regions support enhanced visual performance in early deafness. In early deaf cats, reversible deactivation of posterior auditory cortex selectively impaired their enhanced visual localization performance, while deactivation of the dorsal auditory cortex impacted their visual motion detection, suggesting that it is possible to localize individual visual functions in specific parts of reorganized auditory cortex (Lomber et al., 2010). What is not clear from this study is whether other higher-order brain regions, such as attention networks, also support the enhanced visual performance in deaf animals. Our whole-brain analysis suggests that in humans, other brain regions also support processing of peripheral visual stimuli in deafness. What remains to be elucidated is how these regions interact to support peripheral processing. For example, one TMS-fMRI study in hearing humans reported that FEF imparts unique functional connectivity to primary visual areas representing the periphery (Ruff et al., 2008). This raises the question of whether FEF is an upstream cause of differential anterior visual cortex activation in the deaf or a downstream target of heightened anterior visual cortex activation. Future research using methods such as TMS or resting-state functional connectivity could address the interactions of these regions.

#### Population considerations

A possible confounding variable in visual neuroplasticity studies comparing congenitally deaf to hearing individuals is early acquisition of sign language. Studies that include native hearing signers can separate the neural changes associated with early sign-language acquisition from those linked to auditory deprivation. However, most of the effects that have been attributed to sign language acquisition concern the pattern of hemispheric laterality. Hearing native signers, for example, exhibit motion detection asymmetries favoring the right visual field and temporal-parietal event-related brain potential asymmetries favoring the left hemisphere similar to congenitally deaf participants and opposite to those displayed by hearing non-signing individuals (Neville and Lawson, 1987c; Bosworth and Dobkins, 2002b). Both hearing and deaf native signers viewing moving stimuli also exhibited unique left lateralization of activation in the motion-related brain areas, MT+/V5-MST (Bavelier et al., 2001). Experiments using more complex language-oriented stimuli found unique right-hemispheric activation bias in native deaf and hearing signers that is dependent on the age of acquisition of sign language (Bavelier et al., 1998; Neville et al., 1998; Newman et al., 2001). Data from the current study likely reflects the effect of auditory deprivation rather than sign language acquisition since previous research has demonstrated that hearing native signers do not display the superior motion detection, visual attention, or lower visual field advantages seen in the deaf, nor the enhanced recruitment of A1, MT+/V5, STS, or parietal cortex that deaf individuals do (Neville and Lawson, 1987c; Bavelier et al., 2001; Bavelier and Neville, 2002).

## Conclusion

Our data indicate that the peripheral visual field is an important sensory domain of crossmodal neuroplasticity in the deaf that involves multiple brain regions. These regions range from those classically considered unisensory such as the primary auditory cortex, to higher-level associative areas. Many of the interconnected regions share a functional role in multisensory integration and visuospatial attention. Further research along these lines will contribute fundamental information about the mechanisms, specificity, and constraints of human neuroplasticity. While such information contributes to our basic understanding of neuroplasticity, it can also be harnessed to guide the development and refinement of educational and rehabilitative programs for typically and non-typically developing individuals.

### Conflict of interest statement

The authors declare that the research was conducted in the absence of any commercial or financial relationships that could be construed as a potential conflict of interest.
